# Josh Billings

**Published:** 1885-12

**Authors:** 


### JOSH BILLINGS.

REMINISCENSES OF THE LATE HUMORIST—SPECIMENS OF HIS FUN.

I was sitting down to lunch one morning at Delmonico's with a literary friend when there entered a gaunt, tall man, with stooping shoulders, a slouching walk, iron gray hair and a pair of keen, bright eyes who deposited himself upon a chair at the nearest vacant table. My friend touched my foot to direct my attention to the new comer and softly said: "Do you know who that is?" "No." "That's Josh Billings," he whispered. "I'll ask him to join us."

My friend arose, went to his table, grasped his hand and in another instant I was introduced to the author of a vast amount of misspelled wisdom. "How's every bone in your body?" said Josh to my friend. Then, turning to me, he said: "Glad to see you, sir. Just arrived from England, eh? Bring my cutlet and coffee here," he went on, addressing the waiter, as he inserted his long, lean legs under the table.

Josh rattled on, telling us strings of adventures and now and then uttering a quaint, wise thought. One remark I remember. He was saying that a friend of his had been on a spree for a fortnight and that he had a whisky head on him. "What's that?" I asked. "Why, his head was so swollen that he had to work on his hat with a shoehorn."

Josh was extremely fond of animals and had a cat at his house in Albany which he gravely addressed as "William." I suggested that that was a dignified name for puss, as cats were usually called "Tom" or "Tip" or a quick, short cognomen.

"But that's a special, swell, blue-blooded specimen of the feline race, I wish you to know," rejoined the humorist "Recently, poor fellow, he has had fits, and since then I call him 'Fitz-William.'"

---

Before I left New York I called on Josh Billings with an album and modestly solicited his autograph. He took it on his knees, gave his mouth a comical twist and wrote:

"Thrice is he armed who hath his quarrel just."

THE BARD OF AVON.

"And four times he who gets his blow in fust."

J. BILLINGS.

Mr. Billings," I said, at parting, "it's astonishing how your wise saws and comical straws float all over Christendom. One can scarcely pick up a paper in any part of the world where the English language s spoken but there, in an odd corner, nestles one of your little grains of philosophy. You keep yourself well before the public." His reply was compact, pertinent and to the point: "Yes, sir, I keep myself just sufficiently in the public eye without putting it out."

---

I complained to Josh one September night some years ago, when on this side of the Atlantic, that the nights were so abominably hot I couldn't sleep.

"My dear boy," he replied, "you ought to accustom yourself to these American alterations of heat and cold. Summer and winter I always sleep with three quilts. In summer, I may remark, I put them under me."

---

When Rubenstein was over here he was presented to Josh, and the pianist was careful to impress the American with accounts of the nobility of his ancestors. "My family," said he, loftily, "goes back to the time of the Crusaders. My researches in this direction enabled me to discover that one of my ancestors accompanied the Emperor Barbarossa." Josh smiled, and affecting to be immensely impressed immediately remarked: "On the piano, of course"

---

A good story is told of the humorist being thrown, on one occasion, among a batch of students in a country town near New Haven. He was tramping along with a rusty yellow dog, and entered the barroom of a hotel for some refreshments. A group of the Yale lads chanced to be there on a frolic, and immediately interviewed Billings, whom they evidently mistook for a farmer. They inquired with affected interest after the heal.h of his wife and children, and Josh, with counterfeit simplicity, gave them a graphic account of his family and farm.

"Of course you belong to the church?" asked one of the boys.

"Yes, the Lord be praised, and my father and grandfather before me."

"Now, I suppose you would not tell a lie," said one of the students.

"Not for all the world."

"What will you take for that dog?" pointing to Josh's cur, which was crouching beneath his chair.

"I won't take twenty dollars for that dog."

"Twenty dollars! Why, he's not worth twenty cents."

"I assnre you I would not take twenty dollars for him."

"Come, my friend," said the student, who, with his companions, was bent on having some fun with the old man, "Now, you say you won't tell a lie for the world. Let me see if you will not do it for twenty dollars. I will give you twenty dollars for your dog."

"I'll not take it."

"You will not? Here! let me see if this will not tempt you to a lie," added the student, producing a small bag of half dollars, which he built up into small piles on the table. Josh was sitting by the table, with his hat in his hand, apparently unconcerned. "There," added the student, "there are twenty dollars, all in silver; I will give you that for the animal." Josh quietly raised his hat to the edge of the table, and, as quick as thought, scraped all the money into it except one half dollar, and then exclaimed:

"I won't take your twenty dollars! Nineteen and a half is as much as that dog is worth; he is your property.

A tremendous shout from his fellow students clearly showed the would-be wag that he was completely sold and that he need not look for sympathy from that quarter, so he good-naturedly acknowledged himself beaten.

---

Josh was impatient of the airs and graces of the Boston shop girls. I went with him into a store in Washington street one day, and he asked one of the maidens if she was the attendant who had sold him a handkerchief the day before.

"I am the saleslady who served you," responded the reduced empress in fringed hair and ringed fingers who presided at the counter.

"Well," said Josh, "I will take a dozen more, and as I wish to get them to my was lady at once I will get you to send them to my carriage around the corner. My coach gentleman cannot get to the door just now in consequence of the cart of the ash gentleman blocking the way."

---

It was Josh who originated the phrase that is now a national expression—"The business end of the wasp," and when he said to a lady, "It is better to be laughed at for not being married than to be unable to laugh because you are," it seems to me he uttered a sentence, to use one of his own expressions, "bulging out with first-class wisdom."

---

Three little points of interest before I close. Josh did the greater part of his best work after he was forty years of age, and the *New York Weekly* paid him $100 a week for a column of "Sayings," and he wore the long hair that trailed down his back and flowed over his high coat collar to conceal an unsightly wen on his neck.—*Howard Paul in Philadelphia Times.*

---

THE Japanese cat's-eyes, which are now fashionable ornaments, are the polished hinge or thick knob at the hinge of a pearl oyster.

